# Sympathetic and parasympathetic subtypes of body-first Lewy body disease observed in postmortem tissue from prediagnostic individuals

**DOI:** 10.1038/s41593-025-01910-9

**Published:** 2025-03-13

**Authors:** Katrine B. Andersen, Anushree Krishnamurthy, Mie Kristine Just, Nathalie Van Den Berge, Casper Skjærbæk, Jacob Horsager, Karoline Knudsen, Jacob W. Vogel, Jon B. Toledo, Johannes Attems, Tuomo Polvikoski, Yuko Saito, Shigeo Murayama, Per Borghammer

**Affiliations:** 1 Department of Clinical Medicine, Aarhus University, Aarhus, Denmark; 2 Department of Nuclear Medicine and PET, Aarhus University Hospital, Aarhus, Denmark; 3 Department of Clinical Sciences Malmö, Faculty of Medicine, SciLifLab, Lund University, Lund, Sweden; 4 Stanley Appel Department of Neurology, Houston Methodist, Weill Cornell Medical College, Houston, TX USA; 5 Translational and Clinical Research Institute, Campus for Ageing and Vitality, Newcastle upon Tyne, UK; 6 Cellular Pathology Department, Royal Victoria Infirmary, Newcastle upon Tyne, UK; 7 Brain Bank for Aging Research, Tokyo Metropolitan Geriatric Hospital and Institute of Gerontology, Tokyo, Japan; 8 Brain Bank for Neurodevelopmental, Neurological and Psychiatric Disorders, United Graduate School of Child Development, Osaka University, Osaka, Japan

**Keywords:** Parkinson's disease, Dementia

## Abstract

Recent studies suggest the existence of brain-first and body-first subtypes within the Lewy body disorder (LBD) spectrum, including Parkinson’s disease. These studies primarily focused on α-synuclein propagation through the parasympathetic vagal and olfactory bulb routes, leaving the possibility of a sympathetic nervous system spreading route unexplored. In the present study, we analyzed two postmortem datasets, which included 173 and 129 cases positive for Lewy pathology. We observed a clear distinction between brain-first and body-first subtypes in early prediagnostic cases with mild Lewy pathology. Brain-first cases displayed minimal peripheral organ pathology in prediagnostic phases, contrasting with marked autonomic involvement in prediagnostic body-first cases. Utilizing the SuStaIn machine learning algorithm, we identified two distinct body-first subtypes, one with vagal predominance and another with sympathetic predominance, in equal proportions. Our study supports the existence of three prediagnostic LBD subtypes and highlights the sympathetic nervous system alongside the parasympathetic system in LBD onset and progression.

## Abstract (web summary)

This postmortem study identifies brain-first and two body-first subtypes in prediagnostic cases of Lewy body disease. It highlights sympathetic and parasympathetic pathways in body-first disease, advancing insights on disease onset and progression.

## Main

The aggregation and subsequent cell-to-cell propagation of pathological α-synuclein are theorized as fundamental pathogenic traits in Lewy body disorders (LBDs), including Parkinson’s disease (PD) and dementia with Lewy bodies (DLB)^1–4^. This concept underlies several theoretical disease models and staging systems, which aim to elucidate the origin, evolution and clinical phenotypes of LBD^5–10^. However, these proposed models are mutually incompatible, leaving several critical questions unresolved. First, it is unclear whether Lewy pathology arises unifocally or multifocally and whether the olfactory bulb (OB) and enteric nervous system are indeed the primary sites of origin. Second, most previous human postmortem studies primarily focused on central nervous system (CNS) data, disregarding the peripheral autonomic nervous system. Third, although the gut-to-brain spreading route has been predominantly associated with the vagus nerve, a complementary sympathetic nervous system (SNS) propagation route from the gut via sympathetic connections to the sympathetic trunk remains largely unexplored^5,6^. Finally, in postmortem studies, the emphasis has been chiefly on cases with an antemortem diagnosis of PD or DLB, whereas the earliest stages of Lewy pathology have received limited attention. This is problematic because, at the time of diagnosis, Lewy pathology is already widely disseminated^5,11–16^. With respect to studying the earliest origin sites and initial spreading routes, it is essential to prioritize cases with minimal incidental LBD (ILBD).

In the present study, we address these unresolved questions by analyzing two postmortem datasets, comprising 173 and 129 cases positive for Lewy pathology, most of which were early stage ILBD cases with localized Lewy pathology. Notably, these datasets include tissue samples from both the CNS and the peripheral nervous system (PNS). First, we evaluated the postmortem data using an a priori-based stratification approach based on our brain- versus body-first theoretical disease model^9,17^. The aim was to examine how patterns of cross-sectional postmortem Lewy pathology (rank ordered according to spatial extent and severity) correspond to the spatiotemporal predictions of the brain- versus body-first model. Initially, we divided the postmortem cases into categories according to the extent of disseminated Lewy pathology. We then further stratified them into groups based on plausible brain-first versus body-first etiology: brain-first cases had more Lewy pathology in the amygdala (AMY) and OB compared with the dorsal motor nucleus of the vagus (DMV) and sympathetic trunk (SY), whereas body-first cases displayed the opposite pattern. The plausible site of origin of the first Lewy pathology and the subsequent spatiotemporal evolution of pathology was then examined in 16 different anatomical regions, including the SY and 4 peripheral organs. Finally, we employed the recently developed Subtype and Stage Inference (SuStaIn) machine learning algorithm^18–20^ to examine and potentially verify these progression patterns of Lewy pathology in an unsupervised data-driven fashion.

## Results

### Postmortem data processing, definitions and grouping

**Fig. 1:**
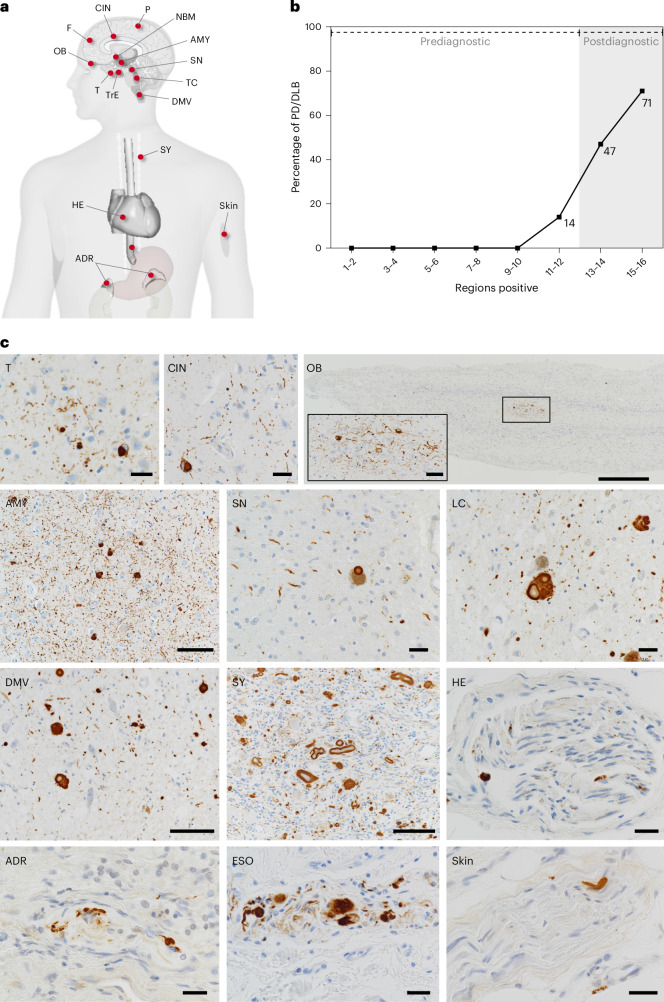
Postmortem data and immunohistochemistry photomicrographs. **a**, A total of 16 anatomical regions staged for Lewy pathology in 173 BBAR cases. **b**, A plot depicting the eight dissemination categories on the *x* axis and the percentage of diagnosed PD or DLB cases on the *y* axis. As diagnosed PD and DLB cases were primarily seen in the two final categories, the first six categories were defined as prediagnostic and the two most disseminated categories were postdiagnostic. **c**, Representative photomicrographs showing phosphorylated α-synuclein immunohistochemical staining in cortical, limbic, brainstem and peripheral tissues. Scale bars, OB 500 µm (insert 50 µm); AMY, DMV and SY 100 µm; all others 25 µm.

For most of the analyses conducted in the present study, we utilized the Brain Bank for Aging Research (BBAR) postmortem dataset^16^. Five ILBD cases were excluded as a result of missing data, resulting in 173 Lewy pathology cases, comprising 44 with antemortem diagnoses of PD (*n* = 8) or PD dementia (PDD) or DLB (*n* = 36) and 129 cases of ILBD. The cohort’s demographic, clinical and neuropathological characteristics are provided in Extended Data Table 1. Immunohistochemistry targeting phosphorylated α-synuclein was applied across 16 anatomical regions, including 4 peripheral organs (heart (HE), adrenal gland (ADR), esophagus (ESO), skin), SY, OB and 10 CNS regions (Fig. 1). Uniform semiquantitative analyses of Lewy pathology for all CNS and PNS regions were performed using the DLB consortium grading system^21^. Scores ranging from 0 (no pathology) to 4 (very severe pathology) were used^16^.

To categorize cases according to the extent of Lewy pathology dissemination, we initially assigned all cases to eight dissemination categories, binned by two-region intervals, ranging from the mildest dissemination category, encompassing cases with Lewy pathology in only one to two regions, to the most disseminated category with pathology observed in fifteen to sixteen regions.

Next, these cases were stratified into brain-first and body-first subgroups, as previously described^10^. To minimize bias, all regions with pathology scores of 4 were converted to 3 before the brain- versus body-first stratification (Methods). Brain-first cases were defined as those having higher average Lewy pathology severity scores in the AMY and OB compared with the DMV and SY (*n* = 69), whereas body-first cases displayed the opposite pattern (*n* = 54). Of cases, 29% (*n* = 50) were labeled indeterminate because their average severity scores were equal in the AMY and OB versus the DMV and SY. Most indeterminate cases (82%) were from the three most disseminated categories, where the AMY, OB, SY and DMV often show severe pathology and thus cannot be separated by the algorithm explained above.

Table 1 summarizes the clinical, demographic and neuropathological data of all cases, stratified according to the eight dissemination categories and brain- versus body-first subgroups.

**Table 1:** Clinical, demographic and neuropathological data of cases stratified into eight dissemination categories

Regions positive	1–2	3–4	5–6	7–8	9–10	11–12	13–14	15–16
**Age (avg) (years)**	83	81	84	82	87	81	86	85
**Cases (*n*)**								
Total	32	20	4	18	9	21	34	35
Brain-first	17	14	2	7	4	10	9	6
Body-first	13	6	2	6	4	5	10	8
Indeterminate	2	0	0	5	1	6	15	21
**PD or DLB (*n*)**								
Total	0	0	0	0	0	3	16	25
Brain-first	0	0	0	0	0	3	4	3
Body-first	0	0	0	0	0	0	4	5
Indeterminate	0	0	0	0	0	0	8	17
**AD (*n*)**								
Total	7	4	1	2	1	5	10	6
Brain-first	7	4	1	2	1	4	4	2
Body-first	0	0	0	0	0	0	1	0
Indeterminate	0	0	0	0	0	1	5	4
**Braak α-syn (avg)**	0.81	2.00	2.75	3.00	3.22	3.57	4.79	5.17
**Braak α-syn (PD or DLB)**	0.00	0.00	0.00	0.00	0.00	5.00	5.13	5.60
**BBAR α-syn (avg)**	0.72	0.83	1.00	0.97	1.22	1.57	3.23	3.48
**NFT (avg)**	2.78	2.75	3.25	2.56	2.78	2.67	3.06	2.51
**SP (avg)**	1.63	1.50	2.00	1.28	1.44	1.48	1.94	1.57
**Clinical stage**	Prediagnostic	Prediagnostic	Prediagnostic	Prediagnostic	Prediagnostic	Mostly prediagnostic	Postdiagnostic	Postdiagnostic

For eight dissemination categories, the following data are listed: average (avg) age, total number (*n*) of cases and numbers of cases (*n*) in brain-first, body-first and indeterminate categories; number of cases with a PD or DLB diagnosis or an Alzheimer’s disease (AD) diagnosis; average Braak Lewy body stage in all cases and in those patient groups with a PD or DLB diagnosis; averaged BBAR, neurofibrillary tangle (NFT), senile plaque (SP) stages.

### Dissemination of pathology in disease stages

Cases with PD or DLB diagnoses comprised 71% of the eighth dissemination category (15–16 regions positive), 47% of the seventh dissemination category (13–14 regions positive), only 14% of the sixth dissemination category (11–12 regions positive) and 0% in the first five dissemination categories (1–10 regions positive) (Table 1). These findings suggest that Lewy pathology is already widely disseminated across most of the PNS and CNS at the time of diagnosis. Therefore, we defined the two final dissemination categories as the postdiagnostic stage of LBD, while considering the first six categories as the prediagnostic stage, encompassing both the earliest symptomless preclinical stages and the later symptomatic prodromal stages, where symptoms such as constipation and rapid eye movement (REM) sleep behavior disorder (RBD) are seen (Fig. 1b).

**Fig. 2:**
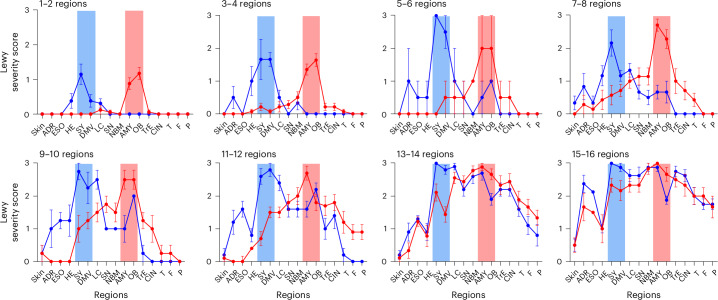
Lewy pathology profiles in dissemination categories. Lewy pathology severity scores of brain-first (red) and body-first (blue) subgroups plotted according to the eight dissemination categories. Blue boxes highlight autonomic structures (the SY and DMV) and red boxes the AMY and OB. Sample sizes of brain- and body-first groups for each dissemination category in each plot are listed in Table 1. Data are plotted as average and s.e.m. TrE, transentorhinal cortex; CIN, anterior cingulum, T, temporal cortex; F, frontal cortex; P, parietal cortex.

Figure 2 shows plots of the brain- and body-first subgroups across the eight dissemination categories. Each plot shows the average pathology score in the 16 anatomical regions for the brain- and body-first subgroups. It is apparent that brain- and body-first groups exhibit distinct patterns of Lewy pathology in the six initial dissemination categories, which gradually converge in the later dissemination categories.

**Fig. 3:**
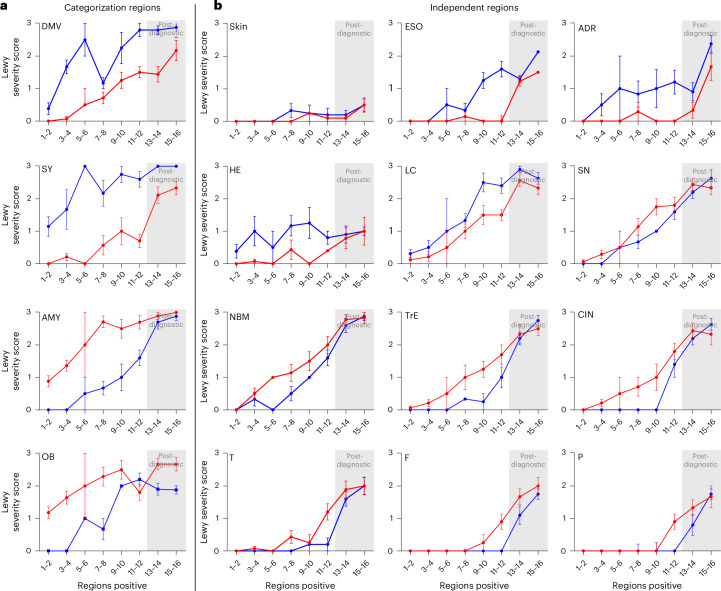
Lewy pathology profiles in 16 anatomical regions. Lewy pathology severity scores of brain-first (red) and body-first (blue) subgroups plotted for each of the 16 anatomical regions examined. **a**, The four regions used for stratifying the brain-first and body-first cases listed in the leftmost column. Those pathology profiles are necessarily biased by the brain- versus body-first stratification procedure. **b**, The other 12 regions not used for stratification that are independent. Body-first patients show peripheral organ pathology at earlier dissemination categories, whereas brain-first patients show earlier limbic or cortical pathology in comparable dissemination categories. Sample sizes of brain- and body-first groups for each dissemination category in each plot are listed in Table 1. Data are plotted as average and s.e.m.

Figure 3 shows the brain- and body-first subgroups for each of the 16 anatomical regions examined. These plots reveal that body-first cases often showed pathology in peripheral tissues during the early prediagnostic dissemination categories. Specifically, the HE and ADR were positive in many body-first cases from the earliest time points. In contrast, brain-first cases showed no or minimal pathology in peripheral tissues across all six initial dissemination categories, with robust peripheral tissue pathology emerging only around the time of diagnosis.

**Fig. 4:**
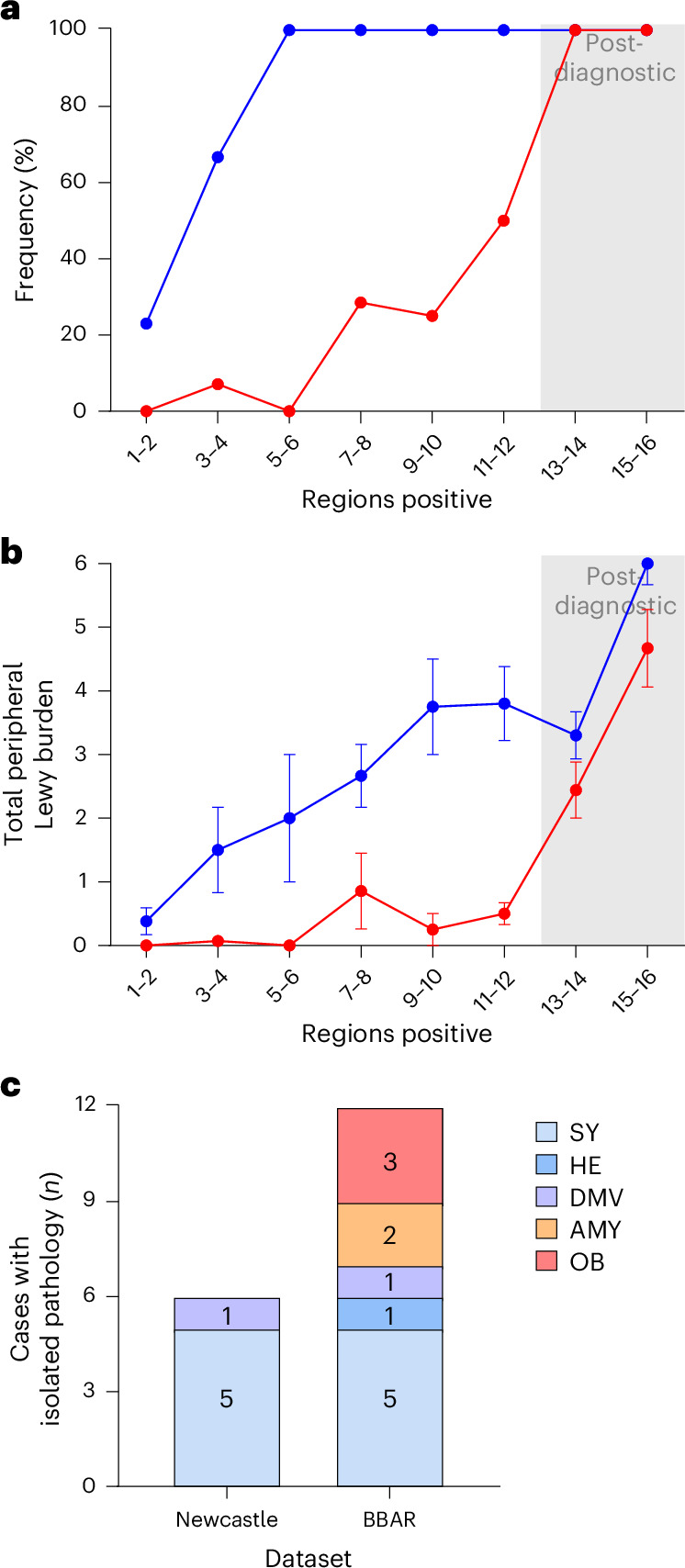
Peripheral organ pathology in brain- and body-first groups. **a**, Frequency of cases showing Lewy pathology in at least one peripheral tissue in brain-first (red) and body-first (blue) groups. Sample sizes of brain- and body-first groups for each dissemination category are listed in Table 1. **b**, Summed Lewy pathology severity scores across the four peripheral organs (HE, ESO, ADR and skin) in brain- and body-first groups. Sample sizes of brain- and body-first groups for each dissemination category are listed in Table 1. Data are plotted as average and s.e.m. **c**, A comparison of the absolute number of cases with confirmed pathology in a single region, within the BBAR and Newcastle datasets. In the Newcastle dataset, no information on OB or AMY was consistently available.

This observation is further emphasized in Fig. 4a, demonstrating that all body-first cases were positive for pathology in at least one peripheral tissue already from the third dissemination category onward. In contrast, brain-first cases did not show robust peripheral pathology until much later, that is, in the sixth dissemination category only 50% were positive. Moreover, the summed Lewy pathology severity scores across the four peripheral tissues were notably higher in body-first cases, indicating that peripheral tissues contain much more Lewy pathology in early body-first disease compared with brain-first disease (Fig. 4b). Thus, body-first cases show marked peripheral pathology early in the prediagnostic phase, whereas brain-first cases do not start to show robust pathology until around the time of diagnosis.

**Extended Data Fig. 1:**
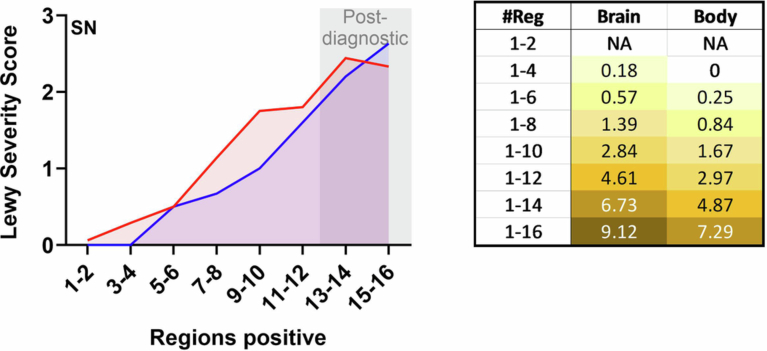
Lewy pathology in substantia nigra of brain- and body-first subgroups. Left. The cumulative area under the curve (AUC) of LBD severity scores is larger in the SN of brain-first (red) than in body-first (blue) LBD in comparable intervals of regions. Right. The AUC values in the table suggest that brain-first cases may be diagnosed with PD based on motor symptoms at earlier dissemination stages compared to body-first, since brain-first cases accumulate more Lewy pathology in the SN at earlier dissemination stages.

The substantia nigra (SN) curves depicted in Fig. 3b and Extended Data Fig. 1 indicate that Lewy pathology arrives in the SN at an earlier dissemination stage in brain-first compared with body-first disease in this dataset. This observation implies that brain-first cases may be diagnosed with PD when Lewy pathology is less disseminated compared with body-first patients. Although speculative, it could mean that the curves from de novo brain-first PD should be shifted approximately one dissemination category to make them comparable to curves from de novo body-first PD, because damage to the SN dictates the time of diagnosis in PD (but not necessarily in DLB). Thus, the frequency of negative peripheral organ biopsies in brain-first PD may be even greater if they are diagnosed one category earlier than what is depicted in Figs. 3 and 4a. It also implies that the levels of cortical pathology will be at similar levels in brain- and body-first PD at the time of diagnosis (bottom row in Fig. 3).

### SNS pathology in early body-first LBD

Although the vagal route of gut-to-brain propagation of Lewy pathology is well established, we and others have argued that a spreading route via the SNS may be of equal importance^9,10,17,22–24^. To test this argument further, we studied an additional postmortem dataset obtained from the Newcastle Brain Tissue Resource (NBTR). This dataset included 129 cases that had been examined in both the stellate (sympathetic) ganglia and CNS regions for the presence of Lewy pathology.

As a result of incomplete staging for Lewy body pathology in relevant CNS regions, our analysis was restricted to cases where data were available for the SY, DMV, locus coeruleus (LC) and SN (*n* = 102).

Among these, we extracted cases showing Lewy pathology in only one single anatomical region. For comparison, we also extracted all such cases with pathology in a single region from the BBAR dataset (Fig. 4c).

In the Newcastle dataset, isolated pathology in a single region was seen in either the DMV (one case) or the SY (five cases). Notably, these six cases were all negative in the AMY, LC and SN. In the BBAR dataset, isolated Lewy pathology in a single region was seen in the DMV (one case), SY (five cases), HE (one case), AMY (two cases) and OB (three cases).

To validate further that the Newcastle cases with isolated pathology in the SY or DMV truly represent isolated pathology across the entire CNS, we crossvalidated the findings using the BBAR dataset. In the BBAR dataset, cases showing Lewy pathology in the SY or DMV but no pathology in the LC, SN or AMY were also always negative for Lewy pathology in all other regions. As the six Newcastle cases shown in Fig. 4c were all negative for Lewy pathology in the LC, SN and AMY, it can be inferred that they were most probably negative for Lewy pathology across the entire CNS, as suggested by the comprehensive BBAR dataset.

In summary, both datasets included five cases with isolated pathology in the peripheral SNS, that is, SY and/or HE, but only one case with isolated pathology in the parasympathetic DMV. This finding strengthens our claim that the sympathetic spreading route could be equally important to the vagal spreading route.

### Subtypes identified by the SuStaIn algorithm

**Extended Data Fig. 2:**
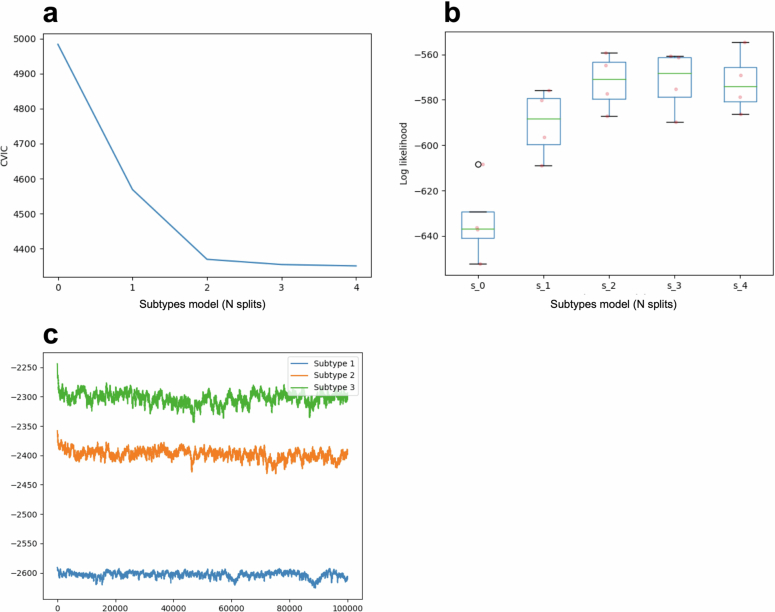
Determination of optimal number of subtypes. **a**. After cross- validation of the complete dataset (score 0-3), the cross-validation information criterion (CVIC) indicates a preferable model fit for 2 N-splits, corresponding to 3 subtypes. **b**. High log- likelihood supports the conclusion that a model with 2 N-splits (3 subtypes) provides the best fit. **c**. Markov Chain Monte Carlo (MCMC) trace supports three different subtypes. The optimal number of subtypes was guided by 10-fold cross-validation. Model uncertainty was estimated through 100.000 Markov Chain Monte Carlo (MCMC) iterations.

The ordinal SuStaIn implementation within PySuStaIn^20^ was applied to the 173 BBAR cases with complete data across 16 anatomical regions. To mitigate potential bias from different Lewy pathology scoring ranges above and below brainstem level (0–4 versus 0–3), all individual instances of regions with a score of 4 were converted to a score of 3 (Methods). A tenfold crossvalidation indicated that three subtypes best supported the model, with minimal additional information gained from adding a fourth subtype This was additionally supported by checking the crossvalidation information criterion (CVIC), log(likelihood) and Markov Chain Monte Carlo (MCMC) trace (Extended Data Fig. 2).

**Fig. 5:**
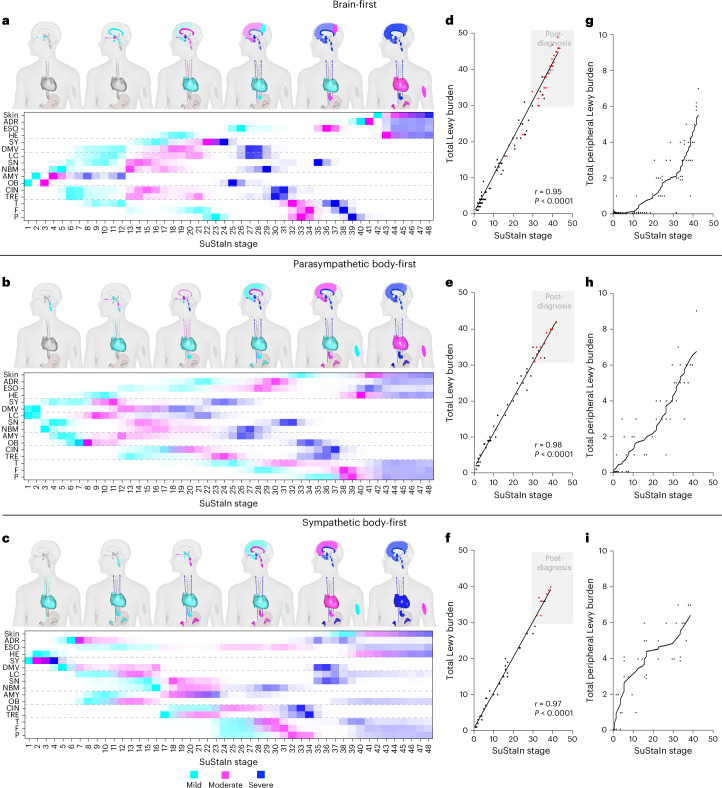
Three subtypes of LBD. The SuStaIn algorithm inferred three different patterns of LBD progression. Maps of Lewy pathology deposition are shown for increasing SuStaIn stages with color codes representing pathology severity (cyan = mild, purple = moderate, blue = severe or very severe). Boxes show the positional variance diagrams, with each box representing the certainty that a region has reached a specific level of pathology at the listed SuStaIn stage, with darker colors representing higher certainty. **a**, A brain-first subtype with disease initiation in the AMY and OB followed by spreading to limbic and upper brainstem regions. Later, the pathology appears in the DMV, SY and cortex. Peripheral tissues start to become positive only when CNS pathology is widely disseminated. **b**, A parasympathetic body-first type, in which the DMV and LC are the first positive regions. The NBM, SN and SY follow next and then the AMY and OB. Peripheral tissue pathology follows soon after and shows robust pathology earlier compared with the brain-first subtype. **c**, A sympathetic body-first type presenting initial pathology in the SY and HE. The SY already shows severe pathology when DMV and ADR pathology appears in SuStaIn stages 5–6. Only later, the LC, AAMY and OB becomes positive followed by the SN and NBM. **d**,**e**,**f**, Pearson’s correlations between SuStaIn stage and total Lewy pathology burden. Diagnosed LBD cases (red dots) are almost exclusively seen above SuStaIn stage 29: brain-first (**d**), parasympathetic body-first (**e**), sympathetic body-first (**f**). **g**,**h**,**i**, Total peripheral pathology across SuStaIn stages: brain-first (**g**), parasympathetic body-first (**e**), ympathetic body-first (**f**). Lines show modeled trajectories calculated using LOESS regression.

The three subtypes were (Fig. 5): a brain-first subtype, a parasympathetic body-first subtype and a sympathetic body-first subtype. The brain-first subtype, encompassing 51% of all cases, was characterized by disease onset in the OB and AMY, followed by subsequent propagation in a rostrocaudal direction. Peripheral organ involvement commenced only after CNS pathology had been widely disseminated (Fig. 5a). The parasympathetic body-first subtype, comprising 27% of all cases, showed initial pathology in the DMV and LC, followed by pathology in the SY, nucleus basalis of Meynert (NBM) and SN, with subsequent AMY and OB involvement (Fig. 5b). The sympathetic body-first subtype, comprising 22% of all cases, showed initial pathology in the SY and HE, followed by pathology in the ADR and DMV. Subsequently, the LC, AMY and OB displayed pathology, followed by the SN (Fig. 5c).

Across all three subtypes, SuStaIn stages strongly correlated with total Lewy burden scores across the 16 regions (Fig. 5d–f). In general, cases with antemortem LBD diagnoses showed the highest SuStaIn stages and total Lewy burden (red data points in Fig. 5d–f). It is interesting that only 2 of 117 cases (1.7%) with a SuStaIn stage <29 had an antemortem LBD diagnosis, whereas 42 of 56 cases (75%) with SuStaIn stages ≥29 had antemortem diagnoses of LBD. This finding suggests the existence of a severity threshold, that is, once the Lewy pathology burden reaches a threshold (in this dataset equivalent to approximately SuStaIn stage 29), the likelihood of considerable motor symptoms and/or dementia leading to clinical LBD diagnoses substantially increases. This finding needs replication in independent cohorts.

We also plotted the total Lewy burden in the four peripheral organs across SuStaIn stages for the three subtypes (Fig. 5g–i). In the brain-first type, the first peripheral pathology typically starts accumulating after SuStaIn stage 20. In the sympathetic body-first type, peripheral pathology accumulated from the earliest SuStaIn stages, with parasympathetic body-first showing an ‘in-between curve’ of accumulation. These findings agree with the differential accumulation of peripheral Lewy pathology seen in the manually constructed brain- versus body-first subgroups (Fig. 4a,b).

**Extended Data Fig. 3:**
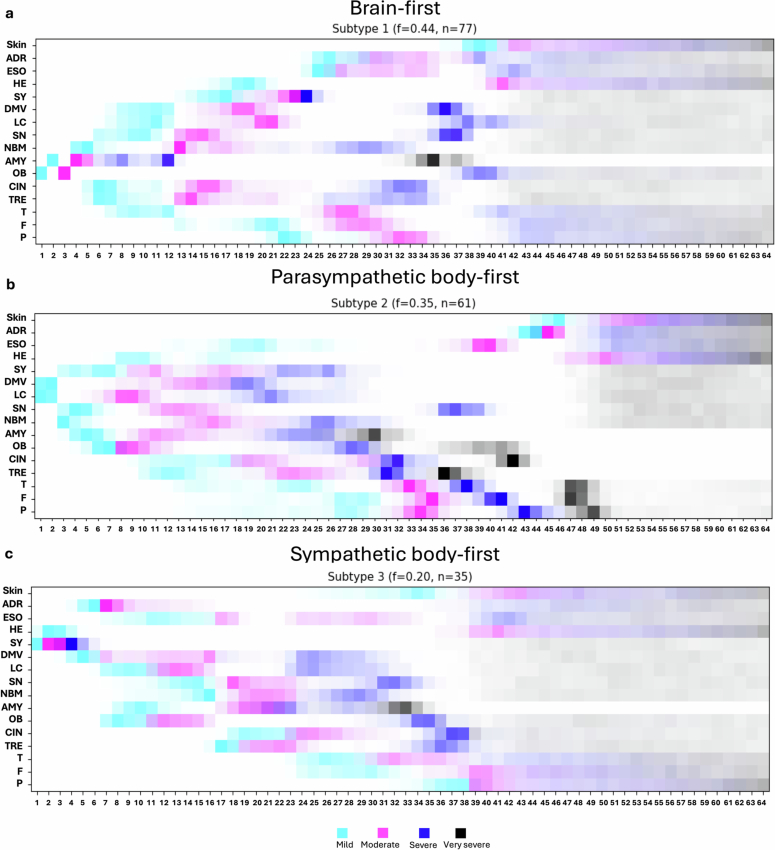
Three subtypes of Lewy body disease. The SuStaIn algorithm inferred three different patterns of Lewy body disease progression. Maps of Lewy pathology deposition are shown for increasing SuStaIn stages with color codes representing pathology severity (red = mild, purple = moderate, blue = severe, black = very severe). Boxes show the positional variance diagrams, with each box representing the certainty that a region has reached a specific level of pathology at the listed SuStaIn stage, with darker colors representing higher certainty. **a**. A brain-first subtype with disease initiation in the amygdala and OB, followed by spreading to limbic and upper brainstem regions. Later, the pathology appears in the DMV, SY and cortex. Peripheral tissues only start to become positive when CNS pathology is widely disseminated. **b**. A parasympathetic body-first type, in which the DMV and LC are the first positive regions. The NBM, SN, and SY follows next and then the amygdala and OB. Peripheral tissue pathology follows soon after and shows robustness much earlier compared to the brain-first subtype. **c**. A sympathetic body-first type presenting initial pathology in the SY and heart. The SY already shows severe pathology when DMV and adrenal gland pathology appears in SuStaIn stage 5-6. Only later, the LC, amygdala and OB becomes positive followed by the SN and NBM.

To further assess the robustness of the SuStaIn analyses, we ran the algorithm on the original dataset, allowing for Lewy pathology scores of 0–4 above the brainstem level. This resulted in three subtypes almost identical to those identified in the analysis utilizing uniform severity stages of 0–3 (Extended Data Fig. 3).

**Fig. 6:**
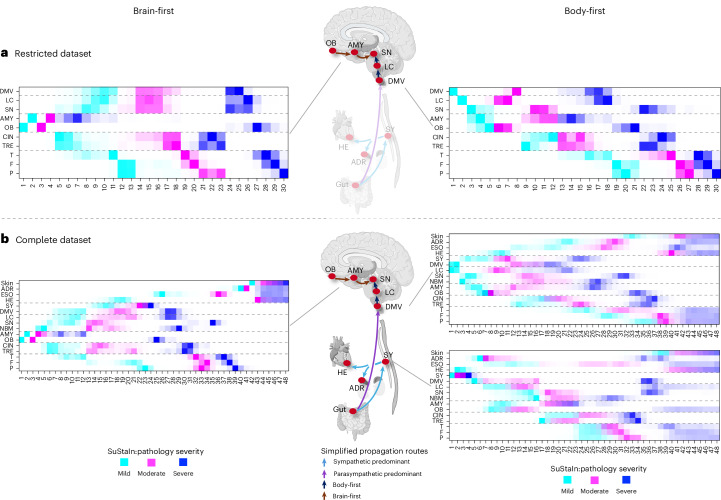
SuStaIn analysis restricted to CNS regions. **a**, Two subtypes suggesting when the SuStaIn analysis was restricted to ten CNS regions. The brain-first subtype (54%) was characterized by initiation in the OB and AMY, followed by propagation to the brainstem and limbic structures, and then the cortical regions. The body-first subtype (46%), characterized by initiation in the DMV followed by caudorostral spreading and involvement of the AMY and OB, occurred only after the LC and SN were involved. **b**, The complete SuStaIn analysis defined one brain-first and two distinct body-first subtypes, as shown here. The arrows in the anatomical figures show simplified theoretical spreading routes as indicated by the SuStaIn positional variance diagrams in the boxes. The diagrams suggest that in parasympathetic-predominant LBD, pathology propagates initially via the vagus (purple arrow), followed slightly later by pathology propagating through the sympathetic connections (blue arrows). In sympathetic-predominant LBD, this order of propagation is reversed. Illustration templates created using BioRender.com.

**Extended Data Fig. 4:**
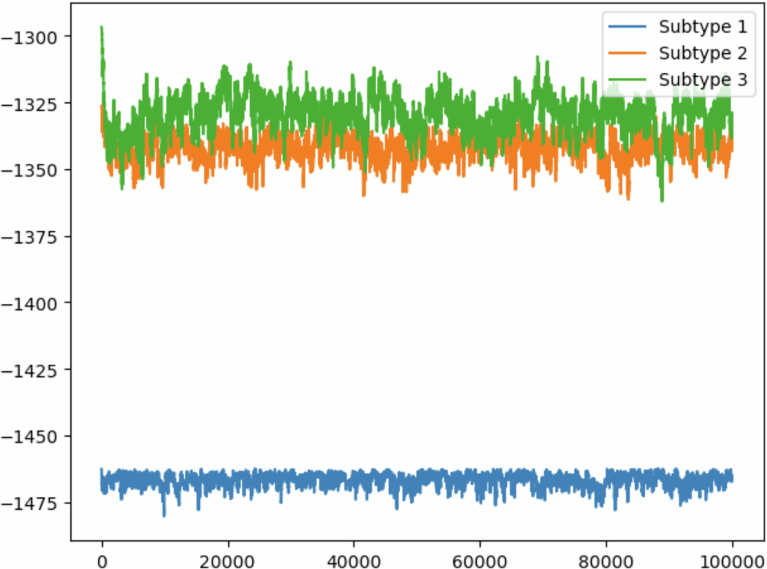
Determination of optimal number of subtypes in restricted dataset analysis. Markov Chain Monte Carlo (MCMC) trace shows that only two sufficiently different subtypes were seen, when restricting the dataset to 10 CNS regions. The optimal number of subtypes guided by 10-fold cross-validation. Model uncertainty was estimated through 100.000 Markov Chain Monte Carlo (MCMC) iterations.

A single study employed SuStaIn to study the progression of Lewy pathology in a different dataset of postmortem cases^25^. However, in that study, only data from ten CNS regions were included in the SuStaIn analysis, without considering the SY or peripheral tissues. Therefore, we conducted a comparable SuStaIn analysis of the BBAR dataset, including the same subset of ten CNS regions (Fig. 6). This restricted analysis yielded only two robust subtypes, each comprising approximately half the cases: a brain-first type characterized by disease initiation in the OB and AMY and a body-first type originating in the DMV with subsequent caudorostral progression in the brainstem. This finding is in broad agreement with the previously published study and emphasizes that, when SuStaIn is applied to a restricted CNS-only dataset, the algorithm is naturally unable to identify the robust sympathetic-predominant subtype, which we detected when using the complete BBAR dataset. The MCMC plot for this restricted analysis is shown in Extended Data Fig. 4.

### Comparison to in vivo imaging data

**Fig. 7:**
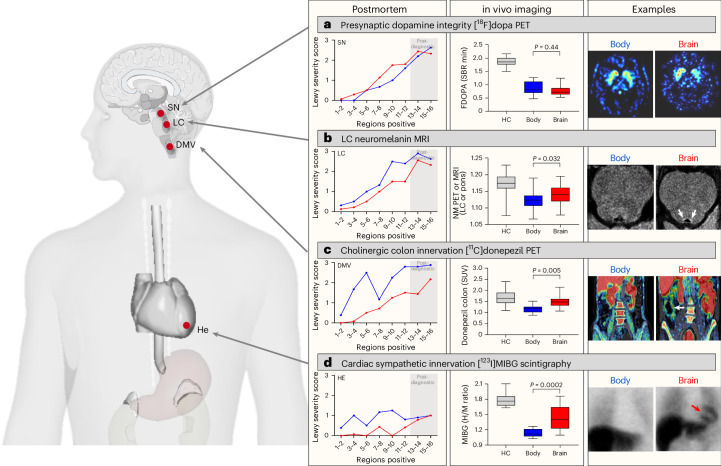
Comparison to multimodal imaging data. Brain- and body-first postmortem subtypes shown alongside previously published multimodal in vivo imaging data from healthy controls (HC), de novo body-first PD with premotor RBD and de novo brain-first PD without premotor RBD^26,27^. **a**, De novo brain- and body-first PD patients showing similar magnitude of dopaminergic denervation at diagnosis (*P* = 0.44). SBR, specific binding ratios. **b**, De novo body-first PD showing reduced neuromelanin density in the LC compared with de novo brain-first PD (*P* = 0.032). The arrows show the LC. **c**, De novo body-first PD showing reduced binding of [^11^C]donepezil in the colon compared with the de novo brain-first PD. This signifies parasympathetic denervation in body-first PD during the prodromal stage (*P* = 0.005). The arrow shows the colon. SUV, standard uptake values. **d**, De novo body-first PD showing severely reduced uptake (invisible heart) of [^123^I]MIBG in the HE compared with de novo brain-first PD (*P* = 0.00019). An invisible heart signifies that cardiac denervation in many cases of prodromal body-first LBD starts ≥10 years before the dopamine system degenerates^44^. The red arrow indicates the heart. The box plots depict the median, 25th and 75th percentiles and the whiskers indicate minimum and maximum values. Sample sizes of all in vivo imaging groups are shown in Extended Data Table 2. Group comparisons of imaging data were conducted using one-way ANOVA with Tukey’s multiple-comparison test. NM, neuromelanin; H/M, heart/mediastinum ratio.

Figure 7 depicts the theoretical relationship between brain- and body-first postmortem subtypes of LBD and corresponding evidence from previously published, multimodal, in vivo imaging studies of these clinical subtypes^26^. The figure summarizes data showing nigrostriatal dopaminergic dysfunction on [^18^F]dopa positron emission tomography (PET; Fig. 7a), reduced neuromelanin signal in the LC on magnetic resonance imaging (MRI; Fig. 7b), cholinergic denervation of the colon on [^11^C]donepezil PET (Fig. 7c) and cardiac denervation on [^123^I]MIBG (*meta*-iodobenzylguanidine) scintigraphies (Fig. 7d). In these clinical studies, body-first PD was defined by the emergence of polysomnography-verified RBD years before diagnosis, whereas brain-first PD did not have RBD at the time of diagnosis. On imaging measures, these de novo body-first patients display evidence of notably more dysfunction of the LC, sympathetic and parasympathetic systems compared with de novo brain-first PD without premotor RBD^26–28^. In addition, de novo body-first patients show increased colonic volume and delayed colonic transit time compared with de novo brain-first patients (Extended Data Table 2)^26,27,29^.

Figure 7 supports body-first patients, who, according to the present postmortem data, accumulate substantial amounts of Lewy pathology in autonomic structures much earlier during the preclinical phase, demonstrating correspondingly more dysfunction and neurodegeneration in these systems on in vivo imaging at the time of diagnosis. Notably, the clinical brain- and body-first groups shown in Fig. 7 were comparable on nigrostriatal dopamine imaging, the burden of motor symptoms and time since diagnosis (Extended Data Table 2).

## Discussion

The main findings of the present study were as follows: (1) at the time of a PD or DLB diagnosis, Lewy pathology is already widely disseminated, affecting most of the vulnerable PNS and CNS regions. (2) Peripheral tissues, including the HE, ADR, ESO and skin, begin to accumulate Lewy pathology very early in the prediagnostic stages of body-first LBD. In contrast, these peripheral organs start to become positive only right before or even after diagnosis in cases with brain-first disease. (3) In body-first LBD, isolated Lewy pathology in the sympathetic PNS is frequent, suggesting that the gut-to-sympathetic propagation route is as important as the vagal gut-to-DMV propagation route. (4) Unsupervised SuStaIn analyses revealed that body-first LBD may comprise two distinct subtypes: a parasympathetic-predominant and a sympathetic-predominant subtype, each accounting for approximately 25% of all LBD cases. In addition, a brain-first subtype of LBD, originating in the OB and AMY, makes up the other half of LBD cases.

Several systems have been proposed for neuropathological staging of LBD^5,7,30,31^. Individual stages in these systems are based on pathology in specific anatomical regions and include only CNS regions. In the present study, we employed an alternative strategy for assessing the neuropathological disease burden across the CNS and the PNS by categorizing the degree of Lewy pathology dissemination from the number of regions involved. Our approach demonstrates that Lewy pathology has already disseminated extensively throughout most of the PNS and CNS at the time of clinical LBD diagnosis. All but three diagnosed LBD cases showed pathology in ≥13 out of the 16 regions investigated. Similarly, all but two cases had SuStaIn stages ≥29. These findings are in accordance with many previous reports, showing that most nondemented PD cases and some prodromal isolated (i)RBD cases are in Braak stages 4–6 postmortem^8,14,32^. Yet, to our knowledge, no studies have conducted a detailed analysis of when the PNS and different peripheral tissues become involved relative to the time of diagnosis. Furthermore, the question of whether such peripheral involvement differs among specific neuropathological subtypes of the disease has also not been explored.

In many brain-first cases, involvement of the peripheral tissues seems to occur in the late prodromal stage (sixth dissemination category) or even postdiagnosis (seventh dissemination category). Indeed, the three diagnosed PD or DLB cases in the sixth dissemination category were classified as brain-first and showed only mild Lewy pathology in the SY and no pathology in the four peripheral tissues. In contrast, body-first cases typically showed peripheral tissue pathology from the earliest time points. From the third dissemination category onward, all body-first cases showed Lewy pathology in at least one peripheral tissue. The cumulative burden of peripheral pathology is already substantial in the early prediagnostic phases of the body-first group, with many single cases showing regions with moderate-to-severe pathology. In addition, our area under the curve analysis of the SN curves suggests that brain-first cases are diagnosed with PD at a less disseminated stage, implying that the prodromal stage could be shorter and that peripheral organs harbor even less peripheral tissue involvement than that depicted^9,23^.

These observations may have implications for diagnostic tools aimed at detecting pathological α-synuclein species in peripheral tissues and biopsies^33–37^. For instance, seed amplification assays (SAAs) have demonstrated impressive diagnostic accuracy in cerebrospinal fluid (CSF)^33,34,36–38^ and promising results have also been reported in blood and biopsies from the skin and gut^35,39,40^. However, these studies predominantly focused on diagnosed PD cases or prodromal LBD cases of the body-first type, characterized by isolated RBD or pure autonomic failure (PAF). At present, we have no solid knowledge about the sensitivity of these methods in prodromal brain-first cases without RBD. The current postmortem findings suggest that such patients could commonly be negative on tests of peripheral tissues even in the late prodromal stage. In support, a recent study reported that only 14% of de novo brain-first PD with normal MIBG scans had positive α-synuclein SAAs in CSF compared with 85% in the group with pathological MIBG^41^. Future studies with optimally sensitive α-synuclein SAAs are needed to determine when patients with brain-first LBD become positive in CSF, blood and peripheral tissues.

Using SuStaIn, we performed an analysis on a large postmortem dataset comprising Lewy pathology-positive cases with high-quality data from the sympathetic ganglia and peripheral tissues. Notably, the BBAR dataset provides a spectrum of Lewy pathology-positive cases from the earliest stages of ILBD to late stage, fully disseminated LBD. The SuStaIn analysis identified three subtypes of LBD. In the parasympathetic-predominant subtype, initial pathology is seen in the DMV and LC, followed by involvement of the SY, SN, NBM and AMY. This pattern is compatible with Lewy pathology originating in the gut and initially spreading via the vagus nerve, before propagating through the SNS.

In the new sympathetic-predominant subtype, pathology emerged earliest in the SY and HE, that is, entirely outside the CNS. This progression pattern fits well with our additional finding that isolated Lewy pathology is frequently observed in the SY and HE. We speculated that this early sympathetic involvement represents Lewy pathology originating in the gut, with preferential spreading through the sympathetic connectome via celiac and mesenteric ganglia to the sympathetic trunk^9,10^. Previous animal studies support this hypothesis, demonstrating that injection of α-synuclein seeds into the duodenum or directly into SY leads to propagation via this sympathetic route^22,42,43^. Of note, in the sympathetic-predominant SuStaIn subtype, Lewy pathology in the SY builds up to severe levels before the involvement of the first CNS structure, typically the DMV.

It is possible that Lewy pathology may linger for an extended period in the SNS of certain LBD cases, leading to considerable build-up and dissemination of pathology throughout the SNS during early disease stages. This proposal could explain the well-documented, severe cardiac sympathetic denervation observed in almost all prodromal body-first LBD, which often begins more than a decade before degeneration of the nigrostriatal dopaminergic system starts^44^. We also speculate that the PAF phenotype may, in many cases, represent a sympathetic-predominant LBD subtype. Patients with PAF show widespread and severe Lewy pathology in skin biopsies exceeding that seen in PD. They also exhibit severe orthostatic hypotension and typically complete loss of cardiac sympathetic innervation, whereas CNS involvement remains limited^35,39,40^. However, many patients who have Lewy pathology-positive PAF progress to RBD and then later PD or DLB on follow-up^45,46^. This time course suggests an underlying etiology with particularly severe but relatively restricted involvement of the SNS during the initial disease stages. Of note, PAF is rare but probably underdiagnosed.

More generally, we speculate that patients with a sympathetic-predominant LBD will often develop a clinical body-first subtype, characterized by marked orthostatic hypotension, peripheral Lewy pathology, cardiac sympathetic denervation and RBD during the prodromal stage. Such cases may sometimes receive a PAF diagnosis, but more commonly they will be diagnosed with iRBD^47^. In an iRBD context, these patients may exhibit autonomic symptoms and signs at the more severe end of the spectrum. In contrast, patients who are iRBD with an underlying parasympathetic-predominant subtype may initially show milder or no orthostatic hypotension, and their cardiac denervation may be less severe compared with that seen in sympathetic-predominant iRBD or PAF. Although almost all cases of iRBD show marked cardiac denervation on MIBG scintigraphies, a range in severity is nevertheless present^26,48^. Also, only ~30% of patients with iRBD have orthostatic hypotension, suggesting a degree of heterogeneity within this prodromal stage of LBD^26,49^. This heterogeneity could partly be explained by the sympathetic- versus parasympathetic-predominant initial spreading of Lewy pathology.

Finally, SuStaIn identified a brain-first group characterized by disease onset in the OB and AMY, followed by rostrocaudal propagation through the brainstem. This subtype, previously labeled limbic-predominant LBD, exhibits relatively late involvement of the sympathetic trunk and peripheral tissues^7,8^.

We found that 33 of the 36 cases with AD diagnoses displayed a brain-first pattern of Lewy pathology, in accordance with previous studies^50^. It is well known that 40–60% of cases of AD show Lewy pathology in the limbic system^51,52^. However, the etiology and potential progression rate to a full-blown LBD in these cases remain unclear. Indeed, postmortem evidence has shown a striking difference in patterns of carboxy-terminal truncations observed between α-synuclein inclusions in the AMY of LBD compared with those seen in patients with AD and mild limbic α-synuclein aggregates^53^. This suggests that the α-synucleinopathy seen in these diseases could be distinct and restricted limbic Lewy pathology in AD may not spread as readily as in classic LBD. Notably, most cases of AD in the present study displayed a brain-first pattern of Lewy pathology. If the Lewy pathology in cases of AD is often self-limiting, this could impact the estimation of the proportion of brain- versus body-first LBD cases, suggesting that body-first etiology is more common in LBD cases without a concomitant AD diagnosis.

The present study has several limitations. First, the cross-sectional nature of postmortem data imposes limitations on the possibility of inferring a temporal evolution of Lewy pathology. Nevertheless, it is possible to assess whether the different stages and patterns of pathology observed in cross-sectional datasets correspond to the predictions of theoretical disease models that incorporate temporal dynamics. The brain- versus body-first model of LBD postulates that Lewy pathology most often arises in either the autonomic system or the OB or AMY, with subsequent spreading via the connectome. The current results, based on cross-sectional data and including the three SuStaIn subtypes, align well with the predictions of this model. Furthermore, the validity of the present results, as well as the brain- versus body-first model in general, is also supported by the complementary patterns seen in in vivo imaging studies of living patients (Fig. 7). Nevertheless, the validity of the proposed subtypes should be further verified by alternative approaches, such as longitudinal studies of prodromal patient cohorts with periodic sampling of tissues from skin, gut and nasal swabs, or by imaging studies using a sensitive α-synuclein PET tracer.

Second, the BBAR dataset represents one of the largest and most comprehensive postmortem datasets with a balanced coverage of PNS and CNS regions across the entire neuroaxis. Nevertheless, the analyses presented in the present paper were biased by the specific regions included in the BBAR data and the degree of granular analysis applied to each region. This bias is particularly important for peripheral tissues. Notably, the BBAR data include an extremely detailed and sensitive study of esophagus pathology, minimizing the risk of false-negative findings in this organ^16^. Also, the frequency of pathology in the heart was higher than that of the esophagus, whereas the frequency of adrenal pathology was only slightly lower. This suggests that false-negative findings may also be rare in these organs. In contrast, a relatively low frequency of positive skin biopsies (only 36% of diagnosed cases of PD or DLB) suggests that the reported profiles of skin pathology may not be fully representative^40^. Thus, it would be expected that mild skin pathology may appear at slightly earlier dissemination categories than reported here. In general, it will be important to replicate the current findings, including the SuStaIn analysis using independent datasets with similar coverage of PNS and CNS regions.

Third, late-stage cases in the BBAR dataset often had pathology scores of 4 in the AMY and OB, whereas brainstem nuclei only showed maximum scores of 3. Thus, we applied a correction before assigning cases to the brain- versus body-first categories (Methods, Supplementary Figs. 1–4 and Supplementary Table 1). This correction did not impact the composition of the brain- and body-first subgroups in the six prediagnostic categories, because 103 of 104 cases (99%) were reassigned to the same subgroups. As all our main findings relate to the prediagnostic phase, they were unaffected by the correction applied. However, in the postdiagnostic categories, 27 of 69 cases (39%) were assigned to a different subgroup after correction. Thus, the proportion of brain- versus body-first cases in the postdiagnostic stage cannot be reliably assessed. This overlap is not surprising because late-stage cases show widespread and severe pathology and the initially distinct brain- and body-first patterns converge on a final shared pattern, where brainstem and limbic regions are approaching saturation.

Fourth, as the Newcastle dataset did not have complete information across the entire CNS, we used the BBAR data to support the validity of the SY-only and DMV-only cases found in the Newcastle dataset. Our findings about the number of cases with isolated pathology in a single region should be reproduced in independent postmortem datasets with full anatomical coverage of CNS and PNS regions.

Finally, different immunohistochemical techniques, the thickness of tissue slices and variable neuropathological staging techniques led to variations in assessing the severity of Lewy pathology. Neuron loss will also impact severity of Lewy pathology in some nuclei at later disease stages. Thus, staging systems and SuStaIn analyses performed on different datasets could yield slightly different trends from those reported here.

In conclusion, we classified LBD cases into brain-first and body-first subtypes on an individual basis. Unsupervised SuStaIn disease progression modeling revealed that body-first LBD can be further divided into sympathetic- versus parasympathetic-predominant subtypes. This comprehensive approach provides new insights into the complex progression of Lewy pathology during its earliest stages. In support of this new finding, we also showed that isolated Lewy pathology in the sympathetic system, without any CNS involvement, is frequently seen among cases at the earliest stages of LBD. Future studies will be needed to validate our findings in vivo in prospective cohorts, using appropriately validated biomarkers, and to elucidate the underlying disease mechanisms and potential triggers responsible for producing these variable subtypes.

By stratifying postmortem data according to the degree of disseminated pathology, we also showed that Lewy pathology is already disseminated throughout most of the PNS and CNS at the time of the LBD diagnosis. Notably, we demonstrate that cases with brain-first LBD typically exhibit minimal involvement of peripheral tissues until right around the time of diagnosis. This observation raises concerns about the sensitivity of diagnosing prodromal brain-first LBD based on skin or other peripheral tissue biopsies. Finally, our findings may have implications for the future biological definitions of LBDs and their subtypes^54,55^.

## Methods

### BBAR dataset

Tissue samples were collected from autopsy cases with consent for the BBAR study^16^. Neuropathological examination was done by board-certified neuropathologists and confirmed by two specialists (Y.S. and S.M.), following the rules of the American Association of Neuropathologists. A total of 518 cases were analyzed (mean age at death 83 ± 9 years), out of which 178 cases had Lewy pathology in at least one anatomical region (any region having a severity score of ≥1). Out of these, 5 cases were removed as a result of incomplete data, leaving 173 cases available for further analysis.

The histological examination involved assessing tissues from 16 different anatomical regions, including 11 regions from the CNS and 5 from the PNS. The CNS regions comprised the DMV, LC, SN, NBM, AMY, TrE, CIN, T, F, P and OB. The PNS encompassed SY, the anterior wall of the left ventricle of the HE, the distal part of the ESO (as a representation of the enteric nervous system), the ADR and the skin of the upper arm and thigh. Half of the PNS tissue and one hemisphere of the CNS tissue were frozen, sliced and preserved for further analysis. The other half (or hemisphere) of the tissues was fixed and sectioned for staining. Staining techniques, including hematoxylin and eosin stain and immunohistochemistry, were utilized for analysis, with additional staining to assess changes associated with aging.

Immunohistochemical staining was performed with the anti-phosphorylated-α-synuclein antibody on 6-μm-thick tissue sections (pSyn#64, 1:20,000, FUJIFILM Wako Pure Chemical Corp.)^56^. To confirm positive immunoreactivity, two anti-phosphorylated-α-synuclein antibodies—rabbit polyclonal antibody (cat. no. PSer129, 1:100, a gift from T. Iwatsubo^57^ and H. Akiyama^58^) and rabbit monoclonal antibody (Abcam, cat. no. MJF-R13, ab168381, 1:80,000)—and an anti-nonphosphorylated-α-synuclein antibody (BioLegend, cat. no. LB509, 1:100; monoclonal, gift from T. Iwatsubo, now available for purchase from BioLegend) were used in select cases posttreatment with proteases. Histological and immunohistochemical evaluations were conducted using light microscopy. Lewy pathology staging was based on BBAR LB stage, DLB Consensus Guidelines and Braak LB stage. Semiquantitative analyses employed the DLB consortium grading system, amyloid-β and phosphorylated tau, Braak senile plaque stage, CERAD score, Thal senile plaque phase, Braak NFT stage and Saito argyrophilic grain stage (Extended Data Table 1).

Semiquantitative severity scores for Lewy pathology were assigned on tissue examination according to the DLB consortium guidelines^19^. All regions were assigned scores ranging from 0 to 4 (0 = no pathological staining; 1 = mild, that is, sparse phosphorylated α-synuclein-immunopositive dots or Lewy neurites (LNs) or diffuse granular cytoplasmic stain in the neuron, neither LBs nor phosphorylated α-synuclein-immunopositive neuronal intracytoplasmic dense aggregations; 2 = moderate, that is, one to three LBs or phosphorylated α-synuclein-immunopositive intracytoplasmic dense aggregations and scattered LNs in a low-power field (×10); 3 = severe, that is, >4 LBs and scattered LNs in low-power field (×10); and 4 = very severe, that is, numerous LBs and LNs with severe immunoreactivity for phosphorylated α-synuclein in the neuropil or background).

The DLB consortium criteria are based on counting absolute numbers of α-synuclein aggregates per low-powered microscopy field. Some anatomical regions, such as the AMY, TrE and OB, often accumulate more extensive Lewy pathology per microscopy field compared with small brainstem nuclei. Thus, in the BBAR dataset, a score of 4 was much more common in the AMY (*n* = 48) or OB (*n* = 27) than in the SY (*n* = 5) or DMV (*n* = 0) in later-stage cases. This discrepancy could heavily bias the subtype stratification algorithm toward brain-first in late-stage cases. To minimize this bias, we therefore converted all scores of 4 to 3, thus producing a corrected dataset with pathology scores ranging from 0 to 3. All results presented in the main paper are based on analysis of the corrected data.

To assess the robustness of our results, analyses were repeated using the original uncorrected data and compared with those from the corrected data. Notably, the assignment of cases to brain-first and body-first subgroups remained robust for the first six dissemination categories. Only 1 case (1%) of the 104 cases across dissemination categories 1–6 was allocated to a different group compared with the corrected data analyses. A comparison of results from the corrected versus uncorrected analyses is summarized in Supplementary Figs. 1–4 and Supplementary Table 1. In brief, all results represented in Table 1 and Figs. 2–4 were almost identical in the two analyses with respect to the first six dissemination categories. Substantial differences were seen only in the postdiagnostic seventh and eighth dissemination categories. This difference did not impact our main results, which are entirely based on findings in the prediagnostic dissemination categories.

### Newcastle dataset

This dataset comprised brain and other tissues donated postmortem to the NBTR by healthy controls or patients with a history of neurodegenerative disease, with approximately half having a specific focus on cerebrovascular stroke. A total of 147 cases are part of this dataset, out of which 129 cases tested positive for Lewy pathology in the SY and/or CNS regions.

We included the 102 NBTR cases with complete information in the SY, DMV, LC and SN. The right hemisphere, brainstem and cerebellum and other tissue samples were immersion fixed in 4% aqueous formalin for 4–6 weeks. Subdissection was performed to obtain routine tissue blocks required to determine neuropathological diagnosis. Tissue blocks were processed through increasing concentrations of alcohol and chloroform before being embedded in paraffin wax. The 6-μm-thick tissue sections underwent antigen retrieval and were incubated with an antibody against α-synuclein (clone KM51, 1:200, Leica). Pathological protein aggregates were visualized using the Menarini X-Cell-Plus HRP Detection Kit with 3,3ʹ-diaminobenzidine as a chromogen. Lewy pathology was assessed according to Lewy body Braak stages and Newcastle McKeith criteria or Lewy pathology consensus criteria^5,30,31^. Similarly, 6-μm-thick tissue sections on stellate ganglia were immunostained for α-synuclein pathology.

Neuropathological staging data were not available across all relevant CNS regions for many of these cases. Nevertheless, this dataset is unique in the sense that it includes tissue samples from the stellate ganglion (SY), which was of particular interest to our analyses. The other regions for which neuropathological staging was relatively complete were the medulla (DMV), pons (LC), midbrain (SN) and CIN. The remaining CNS regions that were sampled, but more infrequently stained, in this dataset were the AMY, entorhinal, OB, NBM, hippocampus, frontal lobe, temporal lobe, occipital lobe, parietal lobe and the motor and sensory areas. For the purpose of our study, we included only those 102 cases that had complete information in the SY, DMV, LC and SN. The neuropathological scoring system involved a binary system of positive or negative pathology, without information about the severity.

### Statistics and visualization

Statistical analyses and data visualization of the semiquantitative data from both datasets were carried out using GraphPad Prism v.10.1.1 (GraphPad Software). LOESS (locally estimated scatterplot smoothing) regression was carried out using R (v.4.3). Human imaging data groups (Fig. 7) were compared using two-sided Student’s *t*-tests.

### SuStaIn disease progression modeling

In our study, we used the ordinal SuStaIn machine learning algorithm to identify new subtypes of LBD and to characterize distinct disease progression trajectories in individual cases. The SuStaIn algorithm is a new methodology, developed by Young et al.^18^ and further refined by Aksman et al.^19^; it integrates concepts from clustering techniques and disease-staging methodologies^18,19^. This algorithm enables the identification of distinct spatial patterns of disease progression by utilizing a data-driven pseudotemporal axis. Moreover, SuStaIn operates on cross-sectional data, making it suitable for our investigation of postmortem datasets.

We employed the ordinal SuStaIn v.1 implementation within PySuStaIn^20^, specifically designed for analyzing categorical data such as neuropathological density scores. Through this approach, we facilitated disease progression modeling using the BBAR dataset described above.

We initially transformed all limbic or olfactory severity scores of 4 to 3. As an input for the algorithm, we then transformed the density scores of 0, 1, 2 and 3 into probabilities by fitting a normal distribution with an s.d. of 0.5 around each score, subsequently normalizing by the sum of probabilities^59^.

Determination of the optimal number of subtypes involved in tenfold crossvalidation was guided by CVIC and log(likelihood). Model uncertainty was estimated through 100,000 MCMC iterations (Extended Data Fig. 2). The model with the lowest CVIC and highest log(likelihood) was selected. Although the algorithm can accommodate multiple progression patterns, our analysis identified three distinct subtypes as optimal. This resulted in the modeling of 48 stages (16 regions × 3 score transitions), representing a gradient in neuropathological scores across 16 distinct regions.

Subsequently, each participant was allocated to a subtype with the most fitting progression pattern and stage^60,61^. The algorithm and analytical procedures utilized in the present study have been comprehensively described in recent studies, ensuring transparency and reproducibility^60,61^.

### Ethics oversight

The research described herein complies with all relevant ethical regulations, including the Declaration of Helsinki. Data from autopsy cases taking place at the Tokyo Metropolitan Geriatric Hospital and Institute of Gerontology (TMGHIG) were obtained with informed consent from all individual participants or their relatives and the study protocol was approved by the institutional ethics review committee of the TMGHIG (2005–16). Data from autopsy cases taking place in Newcastle were obtained with informed consent from participants or relatives and stored within the NBTR, and the study protocol was approved by the Newcastle University Ethics Board (North-East Newcastle & North Tyneside 1 Research Ethics Committee^24^, protocol no. NE 0012). Human imaging data were obtained with informed consent from research participants under protocol no. 1-10-72-195-22 (Regional Science Ethics Committee of Central Region of Denmark). In the imaging studies, patients with PD did not receive financial compensation, adhering to the guidelines from the National Danish Committee on Science Ethics. Healthy controls did receive financial compensation.

### Reporting summary

Further information on research design is available in the Nature Portfolio Reporting Summary linked to this article.

## Online content

Any methods, additional references, Nature Portfolio reporting summaries, source data, extended data, supplementary information, acknowledgements, peer review information; details of author contributions and competing interests; and statements of data and code availability are available at 10.1038/s41593-025-01910-9.

## Supplementary information

Supplementary Information Supplementary Figs. 1–4 and Table 1.

Reporting Summary

## Figures and Tables

**Extended Data Table 1:**
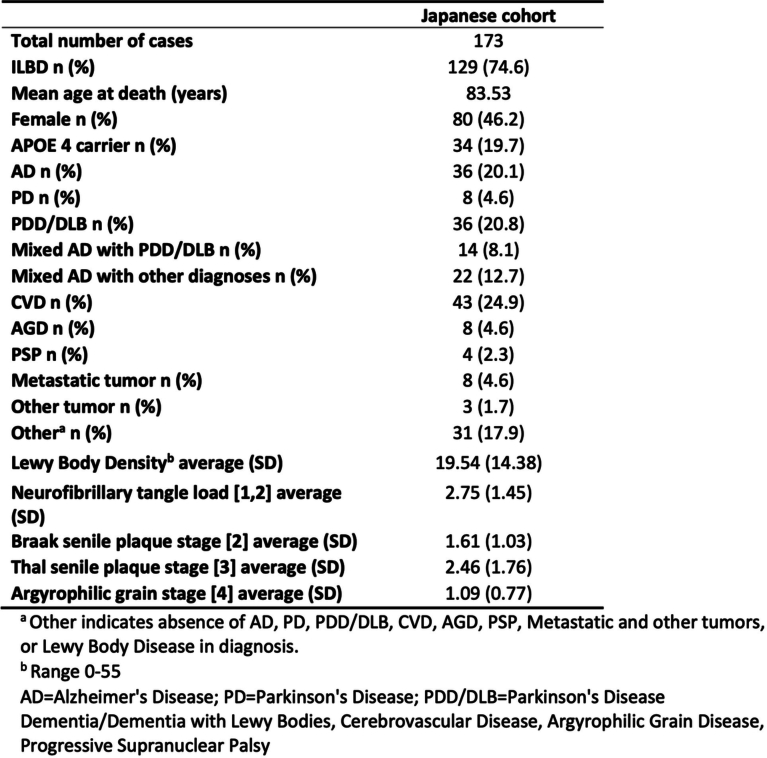
Clinicodemographic and neuropathological data of 173 eligible cases from the BBAR dataset

**Extended Data Table 2:**
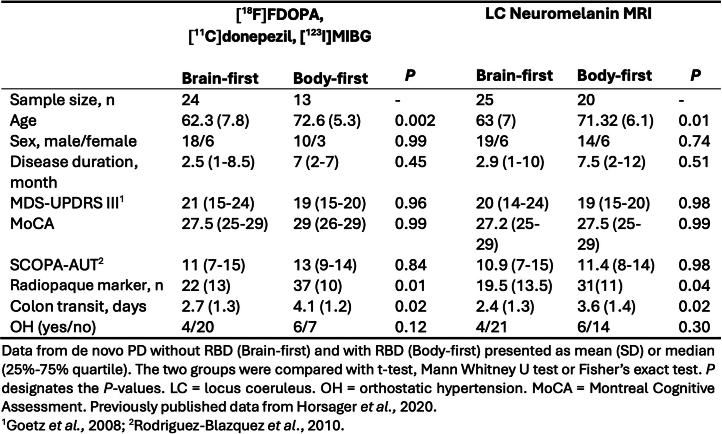
Clinical and demographic information on in vivo imaging data

## Data Availability

The BBAR dataset utilized in this paper has been previously published in full in the supplementary section of ref. ^16^.

## Code availability

All code and notebooks for the SuStaIn algorithm are available through GitHub at https://github.com/ucl-pond/pySuStaIn.
