# Supplementary material for: Sympathetic and parasympathetic subtypes of body-first Lewy body disease observed in postmortem tissue from prediagnostic individuals
Source: Nat Neurosci. 2025 Mar 13;28(5):925–36. doi: 10.1038/s41593-025-01910-9 (PMC12081295; doi:10.1038/s41593-025-01910-9)
Supplement: Supplementary file 1 — Supplementary Figs. 1–4 and Table 1. [file 41593_2025_1910_MOESM1_ESM.pdf]

nature neuroscience

Supplementary information https://doi.org/10.1038/s41593-025-01910-9

Sympathetic and parasympathetic subtypes of body-first Lewy body disease observed in postmortem tissue from prediagnostic individuals

In the format provided by the authors and unedited

SUPPLEMENTARY ONLINE SECTION

The results presented in the main paper was based on analyses of corrected data, where all regions with a semiquantitative pathology score of 4 were converted to 3. The following figures and tables compares results from analyses of the corrected data shown in the main paper with those from uncorrected analyses, where scores of 4 remained in the dataset.

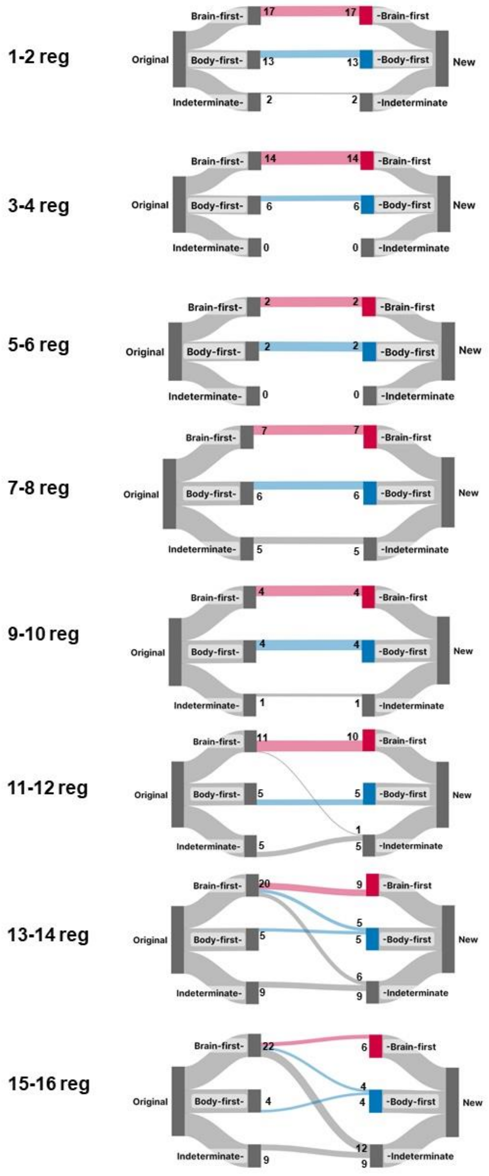

Dissemination category	Total cases	Cases with pathology score 4	Subgroup change in corrected data
1-2	32	0	0
3-4	20	1	0
5-6	4	1	0
7-8	18	1	0
9-10	9	2	0
11-12	21	6	1
13-14	34	21	16
15-16	35	23	20
TOTAL	173	55	37

Supplementary Figure 1. A Sankey diagram showing the case allocation into brain-first, body-first, and indeterminate subgroups in the uncorrected (original) and corrected (new) datasets. No changes in case allocation were seen in the first 5 dissemination categories. In the 6th category a single case changed from the brain-first to the indeterminate group. Thus, among 104 cases in the first 6 categories, only 1 case (1%) were categorized differently. The table shows that in the first 6 categories, only 11 of 104 cases (11%) had a pathology score of 4.

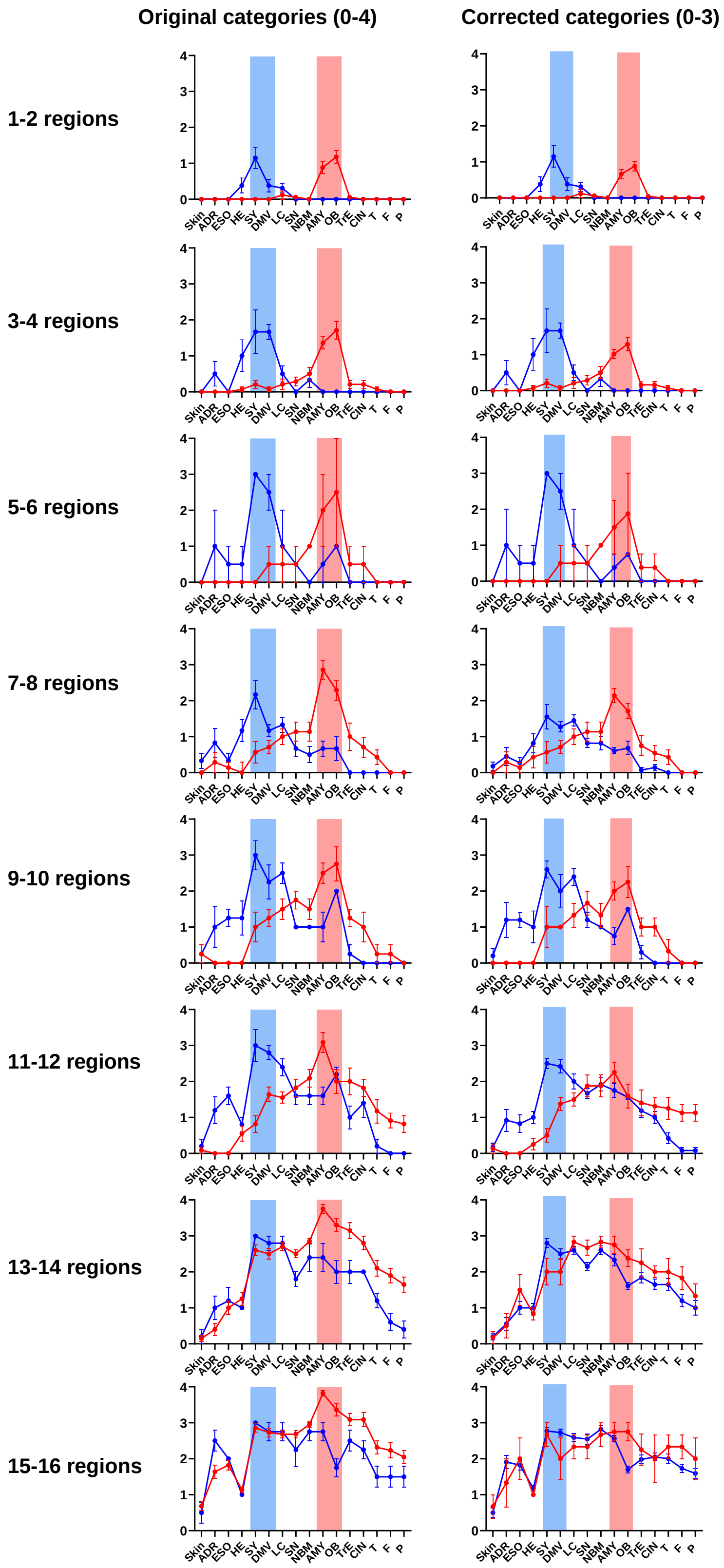

Supplementary Figure 2. A side-by-side comparison of the Lewy pathology profiles in brain- and body-first subtypes. The left column shows results from analyses of original, uncorrected data (score range 0-4). The right column shows the equivalent results from the corrected analysis presented in the revised paper (all scores of 4 converted to 3). As can be seen, the Lewy pathology profiles are almost identical in the first 6 categories in both analyses.

The two final categories (13-14 and 15-16) demonstrate the bias inherent in the original uncorrected data (indicated by *). Notice that the amygdala, OB, transentorhinal cortex (TrE) and cingulum (CIN) have mean Lewy pathology scores between 3 and 4, whereas mean pathology in the brainstem and peripheral tissues do not go above 3. This bias means that most cases will be designated as brain-first in those two final dissemination categories, when using uncorrected data (see Sankey diagram in Supplementary Figure 1). This is not the case for the first 6 categories, where few cases have a score of 4. Data in all graphs are plotted as average and SEM. Sample sizes of the uncorrected data (left column) are listed in supplementary Table 1. Sample sizes of the corrected data are listed in Table 1 of the manuscript.

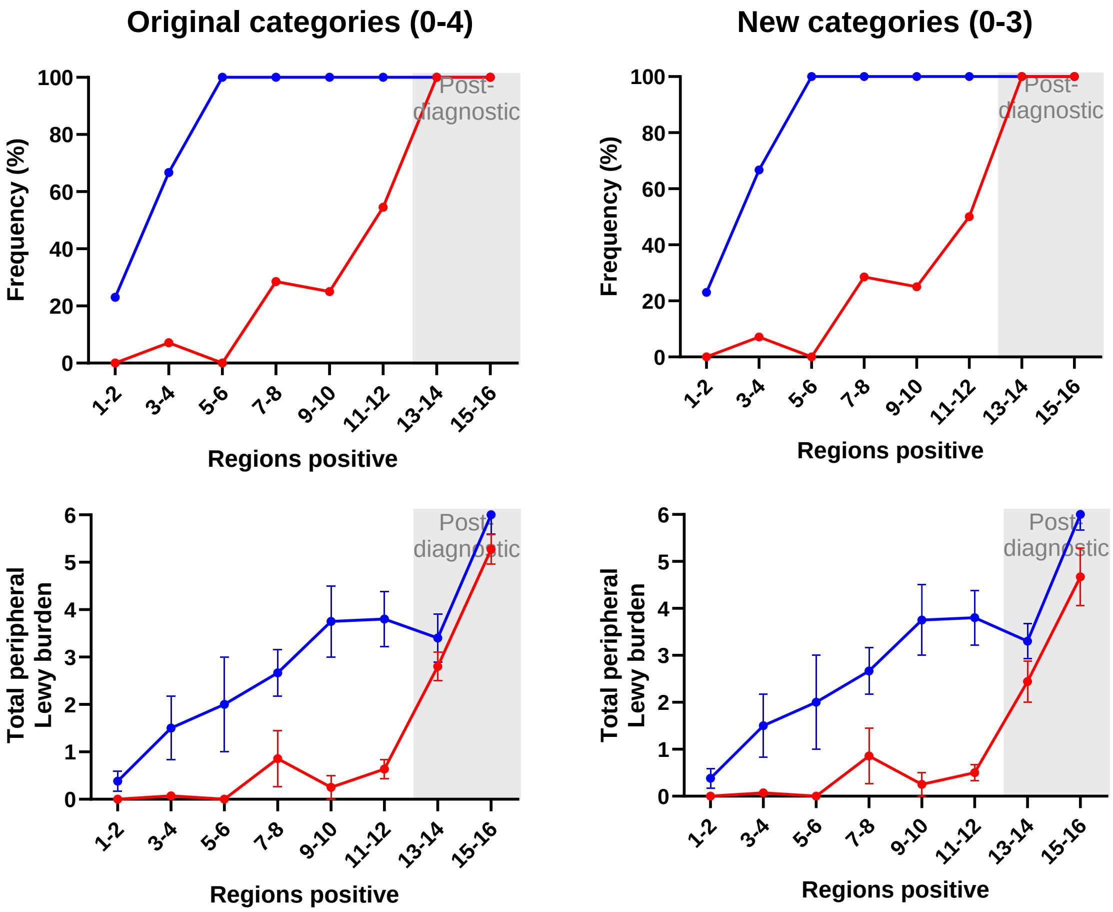

Supplementary Figure 3. A side-by-side comparison of the frequency of cases with Lewy pathology in at least one peripheral region (top row) and the total burden of peripheral Lewy pathology in four peripheral tissues (bottom row). The figure shows that the profiles in brain-first (red) vs. body-first (blue) groups were very similar irrespective of whether uncorrected (left column) or corrected (right column) data were used. Both analyses show that the frequency and total burden of Lewy pathology is much higher in body-first cases, and that brain-first cases do not show robust pathology until close to the time of diagnosis. Data in the bottom two graphs are plotted as average and SEM. Sample sizes of the uncorrected data (left column) are listed in supplementary Table 1. Sample sizes of the corrected data are listed in Table 1 of the manuscript.

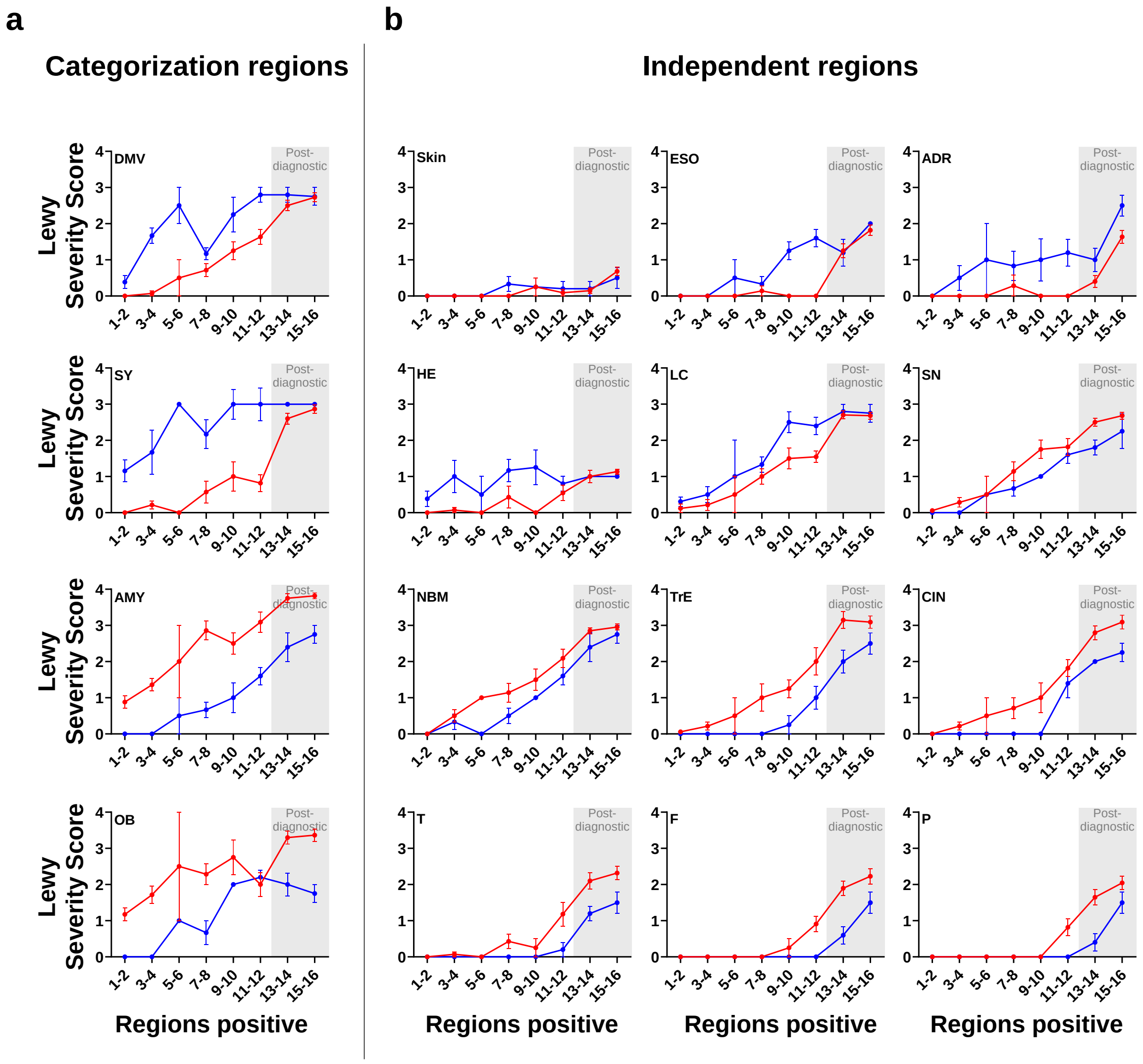

Supplementary Figure 4. Lewy pathology severity scores of brain-first (red) and body-first (blue) subgroups derived from analyses of the uncorrected data. The different profiles of progressive pathology in brain-first (red) and body-first (blue) groups were very similar to the profiles seen in the analyses of corrected data (compare to Figure 3 in the main paper). Data in all graphs are plotted as average and SEM. Sample sizes are listed in supplementary Table 1.

Supplementary Table 1. Clinical, demographic, and neuropathological data in the eight dissemination categories based on analyses of uncorrected data (semiquantitative Lewy pathology scores 0-4).

Regions positive	1-2	3-4	5-6	7-8	9-10	11-12	13-14	15-16
**Age (Avg)**	83	81	84	82	87	81	86	85
**Cases (n)**								
Total	32	20	4	18	9	21	34	35
Brain-first	17	14	2	7	4	11	20	22
Body-first	13	6	2	6	4	5	5	4
Indeterminate	2	0	0	5	1	5	9	9
**PD/DLB (n)**								
Total	0	0	0	0	0	3	16	25
Brain-first	0	0	0	0	0	3	11	17
Body-first	0	0	0	0	0	0	1	1
Indeterminate	0	0	0	0	0	0	4	7
**AD (n)**								
Total	7	4	1	2	1	5	10	6
Brain-first	7	4	1	2	1	4	9	5
Body-first	0	0	0	0	0	1	0	0
Indeterminate	0	0	0	0	0	0	1	1
**Braak α-syn (Avg)**	0.81	2.00	2.75	3.00	3.22	3.57	4.79	5.17
**Braak α-syn (PD/DLB)**	0.00	0.00	0.00	0.00	0.00	5.00	5.13	5.60
**BBAR α-syn (Avg)**	0.72	0.83	1.00	0.97	1.22	1.57	3.23	3.48
**NFT (Avg)**	2.78	2.75	3.25	2.56	2.78	2.67	3.06	2.51
**SP (Avg)**	1.63	1.50	2.00	1.28	1.44	1.48	1.94	1.57
**Clinical Stage**	Pre-diagnostic	Pre-diagnostic	Pre-diagnostic	Pre-diagnostic	Pre-diagnostic	Mostly pre-diagnostic	Post-diagnostic	Post-diagnostic
