# Supplementary material for: Sympathetic and parasympathetic subtypes of body-first Lewy body disease observed in postmortem tissue from prediagnostic individuals
Source: Nat Neurosci. 2025 Mar 13;28(5):925–36. doi: 10.1038/s41593-025-01910-9 (PMC12081295; doi:10.1038/s41593-025-01910-9)
Supplement: Supplementary file 2 — Reporting Summary [file 41593_2025_1910_MOESM2_ESM.pdf]

nature portfolio

Corresponding author(s): Per Borghammer

Last updated by author(s): Jun 10, 2024

Reporting Summary

Nature Portfolio wishes to improve the reproducibility of the work that we publish. This form provides structure for consistency and transparency in reporting. For further information on Nature Portfolio policies, see our Editorial Policies and the Editorial Policy Checklist.

Statistics

For all statistical analyses, confirm that the following items are present in the figure legend, table legend, main text, or Methods section.

n/a	Confirmed	
☐	☒	The exact sample size (*n*) for each experimental group/condition, given as a discrete number and unit of measurement
☒	☐	A statement on whether measurements were taken from distinct samples or whether the same sample was measured repeatedly
☐	☒	The statistical test(s) used AND whether they are one- or two-sided<br>*Only common tests should be described solely by name; describe more complex techniques in the Methods section.*
☒	☐	A description of all covariates tested
☐	☒	A description of any assumptions or corrections, such as tests of normality and adjustment for multiple comparisons
☐	☒	A full description of the statistical parameters including central tendency (e.g. means) or other basic estimates (e.g. regression coefficient) AND variation (e.g. standard deviation) or associated estimates of uncertainty (e.g. confidence intervals)
☐	☒	For null hypothesis testing, the test statistic (e.g. *F*, *t*, *r*) with confidence intervals, effect sizes, degrees of freedom and *P* value noted<br>*Give P values as exact values whenever suitable.*
☒	☐	For Bayesian analysis, information on the choice of priors and Markov chain Monte Carlo settings
☒	☐	For hierarchical and complex designs, identification of the appropriate level for tests and full reporting of outcomes
☐	☒	Estimates of effect sizes (e.g. Cohen's *d*, Pearson's *r*), indicating how they were calculated

Our web collection on statistics for biologists contains articles on many of the points above.

Software and code

Policy information about availability of computer code

Data collection	No software was used for data collection.
Data analysis	All code and notebooks for the SuStaIn algorithm are available through GitHub at https://github.com/ucl-pond/pySuStaIn. We used SuStaIn version. We used the ordinal SuStaIn v. 1.0 (Young et al. Frontiers in AI 2021).<br>Software packages used for other statistical analyses and visualization include the following:<br>Graphpad Prism v 10.1.1<br>R (The R project for statistical computing) version 4.3

For manuscripts utilizing custom algorithms or software that are central to the research but not yet described in published literature, software must be made available to editors and reviewers. We strongly encourage code deposition in a community repository (e.g. GitHub). See the Nature Portfolio guidelines for submitting code & software for further information.

Data

Policy information about availability of data

All manuscripts must include a data availability statement. This statement should provide the following information, where applicable:

- Accession codes, unique identifiers, or web links for publicly available datasets
- A description of any restrictions on data availability
- For clinical datasets or third party data, please ensure that the statement adheres to our policy

The BBAR dataset utilized in this paper has been previously published in full in the supplementary section of Tanei et al., 2021 (ref 15).

Research involving human participants, their data, or biological material

Policy information about studies with human participants or human data. See also policy information about sex, gender (identity/presentation), and sexual orientation and race, ethnicity and racism.

Reporting on sex and gender	No sex-specific analysis is reported in this study. A summary of sex breakdown is found in Extended data Table 1. Unaggregated numbers are available upon request.
Reporting on race, ethnicity, or other socially relevant groupings	No data on race, ethnicity or other relevant social groupings are provided in this study.
Population characteristics	All postmortem samples were from patients with Lewy pathology present in the brain or peripheral tissues. Full breakdown of exact numbers, sex, age, and other parameters are found in Table 1 and Extended data table 1. In vivo imaging data were from patients with Parkinson's disease or from healthy control subjects. Full breakdown of data is provided in Extended data table 3.<br>No data on race, ethnicity or other relevant social groupings are provided in this study.
Recruitment	All postmortem specimens used in this study were obtained with informed consent from the patients or relatives. All in vivo imaging data from patients or healthy control research subjects were obtained with informed consent.
Ethics oversight	The research described herein complies with all relevant ethical regulations, including the Declaration of Helsinki. The postmortem data and the human imaging data were obtained with informed consent. Data from autopsy cases taking place at Tokyo Metropolitan Geriatric Hospital and Institute of Gerontology (TMGHIG) were obtained with informed consent from all individual participants or their relatives and the study protocol was approved by the institutional ethics review committee of the TMGHIG (2005-16). Data from autopsy cases taking place in Newcastle were obtained with informed consent from participants or relatives and stored within the Newcastle Brain Tissue Resource, and the study protocol was approved by the Newcastle University Ethics Board (The North-East Newcastle & North Tyneside 1 Research Ethics Committee; ref 24 NE 0012). Human imaging data were obtained with informed consent from research participants under protocols 1-10-72-195-22 (Regional Science Ethics Committee of Central Region of Denmark). In the imaging studies, patients with Parkinson's disease did not receive financial compensation adhering to the guidelines from the National Danish Committee on Science Ethics. Healthy controls did receive financial compensation. Brain donors in the Tokyo Brain Bank for Aging Research did not receive financial compensation.

Note that full information on the approval of the study protocol must also be provided in the manuscript.

Field-specific reporting

Please select the one below that is the best fit for your research. If you are not sure, read the appropriate sections before making your selection.

☒ Life sciences ☐ Behavioural & social sciences ☐ Ecological, evolutionary & environmental sciences

For a reference copy of the document with all sections, see nature.com/documents/nr-reporting-summary-flat.pdf

Life sciences study design

All studies must disclose on these points even when the disclosure is negative.

Sample size	We used all eligible patient datasets from the Brain Bank for Ageing Research (BBAR) study. The total dataset included 178 cases, but we excluded 5 cases with missing data (i.e. some anatomical regions not examined), leaving 173 cases with complete datasets. We used all eligible patients from the Newcastle Brain Tissue Resource. The total number of Lewy-positive cases was 129, but some cases had missing data. We included the subset of cases (n=102) with complete data in the brainstem regions and sympathetic trunk (explained in the methods section). The human imaging data (Figure 7) has been previously published. Sample sizes are listed in Extended data Table 2. The in vivo imaging data are shown in this paper to put the novel discoveries of the postmortem data into context. The sample sizes of those human imaging data were based on our own sample size calculations, when we performed that study. The imaging data is published (Horsager et al., Brain 2020), which is mentioned in the methods section and in the figure legends.[18F]FDOPA PET of the dopamine system and [123I]MIBG SPECT of the cardiac system is used throughout the world as diagnostic tools, since they work at the individual patient level (n=1) to determine if a patient shows signs of neurodegeneration in the dopamine or cardiac sympathetic system.<br>Concerning [11C]donepezil PET of the intestinal parasympathetic innervation, we had performed several previous studies on groups of

Parkinson's patients and healthy controls and repeatedly shown that significant difference s can be detected with group sizes 12 PD patients in the moderate disease stage vs. 12 healthy controls (Gjerløff et al., Brain 2015) and in 19 PD patients in the early disease stage vs. 16 controls (Fedorova et al., Neurology 2017). No other group has published on donepezil PET of the gut, so we had only our own data with respect to estimating sample sizes and power-
Finally, about neuromelanin MRI of the locus coeruleus, several groups have shown that 12-25 subjects in each group is sufficient to demonstrate statistically significant differences, e.g. Garcia-Lorenzo et al., Brain 2013, Ehrminger et al., Brain 2016, Knudsen et al., Lancet Neurol 2018 (our paper), Sommerauer et al., Brain 2018 (our paper).

Data exclusions	As described above, in the BBAR dataset, we excluded cases with any kind of missing neuroanatomical staining data (n=5). In the Newcastle postmortem dataset, we excluded 27 cases with important missing data, i.e. missing immunohistochemical data from brainstem regions or the sympathetic trunk.
Replication	External replication was not possible since there are no equivalent postmortem datasets available, which includes both full coverage of the CNS regions included in a standard neuropathological assessment of Lewy pathology plus the sympathetic trunk and several peripheral organs. Of note, in the paper we included the Newcastle dataset, which in itself constitutes an external validation of the BBAR dataset. In the BBAR dataset, we found that more subjects had isolated pathology in the sympathetic trunk (n=5) compared to the DMV (n=1). Our finding in the Newcastle dataset replicated this finding, i.e. isolated pathology in the sympathetic trunk (n=5) vs. the DMV (n=1).
Randomization	This was an observational study from postmortem human tissues. Therefore, subjects were not randomized into outcome groups.
Blinding	This was an observational study on all available and eligible postmortem data from two datasets. Thus, the investigators were not blinded to group allocation.

Reporting for specific materials, systems and methods

We require information from authors about some types of materials, experimental systems and methods used in many studies. Here, indicate whether each material, system or method listed is relevant to your study. If you are not sure if a list item applies to your research, read the appropriate section before selecting a response.

Materials & experimental systems

n/a	Involved in the study	
☐	☒	Antibodies
☒	☐	Eukaryotic cell lines
☒	☐	Palaeontology and archaeology
☒	☐	Animals and other organisms
☒	☐	Clinical data
☒	☐	Dual use research of concern
☒	☐	Plants

Methods

n/a	Involved in the study	
☒	☐	ChIP-seq
☒	☐	Flow cytometry
☒	☐	MRI-based neuroimaging

Antibodies

Antibodies used	In the BBAR dataset, the primary antibody used was an anti-phosphorylated-α-synuclein antibody pSyn#64 on 6 µm thick tissue sections (pSyn#64, 1:20,000, FUJIFILM Wako Pure Chemical Corporation, Japan). In the Newcastle dataset, another primary antibody against α-syn was used (KM51 clone, 1:200. Leica, UK).
Validation	In studies where immunohistochemical staining was performed with the anti-phosphorylated-α-synuclein antibody pSyn#64 on 6 µm thick tissue sections (pSyn#64, 1:20,000, FUJIFILM Wako Pure Chemical Corporation, Japan), positive immunoreactivity was confirmed with two anti-phosphorylated-α-synuclein antibodies—rabbit polyclonal antibody (PSer129, 1:100) and rabbit monoclonal antibody (MJF-R13, ab168381, 1:80,000). Additionally, anti-non-phosphorylated-α-synuclein antibody (LB509, 1:100) were used in select cases post-treatment with proteases. Full details available in Tanei et al., 2021 (ref 15).

Plants

Seed stocks	NA
Novel plant genotypes	NA
Authentication	NA
